# Some Virulence Factors of Staphylococci Isolated From Wound and Skin Infections in Shahrekord, IR Iran

**DOI:** 10.5812/jjm.9225

**Published:** 2014-04-01

**Authors:** Azizollah Ebrahimi, Maryam Ghasemi, Bahram Ghasemi

**Affiliations:** 1 Institute of Zoonotic Diseases, School of Veterinary Science, Shahrekord University, Shahrekord, IR Iran; 2 School of Veterinary Science, Shahrekord University, Shahrekord, IR Iran; 3 Tous Hospital, Tehran, IR Iran

**Keywords:** Staphylococci, Skin infections, Virulence Factors, Antibiogram

## Abstract

**Background:**

Staphylococci release a large number of enzymes. Some of these, such as coagulase, beta- lactamase, hemolysins and biofilms are considered indices of pathogenicity.

**Objectives:**

The aim of the current study was based on the isolation and identification of* Staphylococcus aureus* and coagulase negative Staphylococci (CNS) strains from various skin lesions and examining their biofilms, beta- lactamase, hemolysins production and antibiotic resistance pattern.

**Materials and Methods:**

Sixty one infected wounds and 39 skin infections samples were collected and examined. After the culture and identification, examination for production of hemolysins, beta- lactamase, biofilm and susceptibility toward 9 antimicrobials was performed.

**Results:**

Out of 75 isolated Staphylococci, sixty (80%) were biofilm producers. Two overall prevalence of 28.5% and 100% of ß-lactamase production were recorded for isolated *S. aureus* and CNS, respectively. Twenty out of 49 (40.8%), the same number of α- and β- hemolytic *S. aureus*, were isolated while six (12.24%) were ∂ -hemolysin producers. Twenty two of Twenty six (84.6%) isolates of CNS, were hemolysin producers that all were ∂ type. The *S. aureus *isolates from wound infections, show a high sensitivity pattern to all examined antibiotics, this sensitivity pattern for isolates from skin dermatitis is relatively low, though.

**Conclusions:**

High percentage of hemolysins, biofilm and beta lactamase production by isolated Staphylococci, suggests an important role of these virulence factors in the pathogenesis of isolated Staphylococci from dermatitis lesions. The *S. aureus *isolates from wound infections, show a high sensitivity pattern to all examined antibiotics. Only ciprofloxacin was found to be active against all isolates from dedermatitis lesions.

## 1. Background

Skin and skin-structure infections are often caused by Staphylococci or streptococci (1). *Staphylococcus aureus* skin infections were classified as primary or secondary. Primary infections were those occurring on apparently normal skin, and mainly comprised impetigo, ecthyma, folliculitis, furuncles, sycosis barbae, cellulitis, abscesses, paronychia and whitlows. Secondary infections were those arising in damaged skin (traumatized skin, or a pre-existing skin disease) (2).

Coagulase-negative Staphylococci (CNS) are mainly important component of the normal skin flora. From the time of the early 1980s, CNS have also emerged as an important pathogen (3). Staphylococci release a large number of enzymes. Some of these, such as coagulase, hemolysins and proteinases are considered indices of pathogenicity. The first step in the establishment of an infection is the attachment of bacteria to tissues. The production of an abundant glycocalyx by *S. aureus* cells are the minimum requirement of the production of biofilm (4) that is another index of pathogenicity. Currently, a clinically significant number of staphylococcal species that infects humans and domestic animals, exhibits some degree of antimicrobial resistance. The best known mechanism of bacterial resistance is resistance to ß-lactam. Therefore, empiric therapy for suspected staphylococcal infections should always include a ß-lactamase stable antibiotic.

## 2. Objectives

The aim of the current study was based on the isolation and identification of *S. aureus* and CNS strains from various skin lesions and examining their biofilms, beta -lactamase and hemolysins production. Also antibiotic resistance pattern of isolates was examined.

## 3. Materials and Methods

### 3.1. Collection of Samples

From April 2010 to April 2011, Sixty one non-surgical traumatic wound infections and surgical site infections in Emam Ali Hospital patients in Shahrekord, and 39 skin infections (Acne) samples from people referred to dermatology clinics, were collected. Microbiological culture from each lesion was obtained aseptically using a sterile wet cotton swab during the visit and transported to the microbiology laboratory in Stuart transport medium, (Quelab cat.QB-65-5015). In laboratory, the swabs were inoculated in tryptic soy broth (TSB), (Merck, Germany), and incubated at 37°C for 24 hrs. Specimens from incubated TSBs were plated on 5% sheep blood agar (BA), (Merck, Germany), incubated at 37°C and examined daily for 3 days. Colonies typical of Staphylococci, were selected and followed by further examinations.

### 3.2. Identification of *Staphylococcus* Species

Colonies growing on BA were streaked on freshly prepared plates of mannitol salt agar (MSA) and incubated again. Primary characterization of isolates was based on the Gram stain, morphological and cultural characteristics. Colonies were tested with slide coagulase (using rabbit plasma) and DNase test. The catalase and oxidase tests were followed by biochemical examinations according to Murray et al (5). The isolates were kept frozen at −70 °C in Tryptose soy broth containing 15% (v/v) glycerol, until the further examinations were carried out. 

### 3.3. Essay for Hemolytic Activity

**Figure 1.**
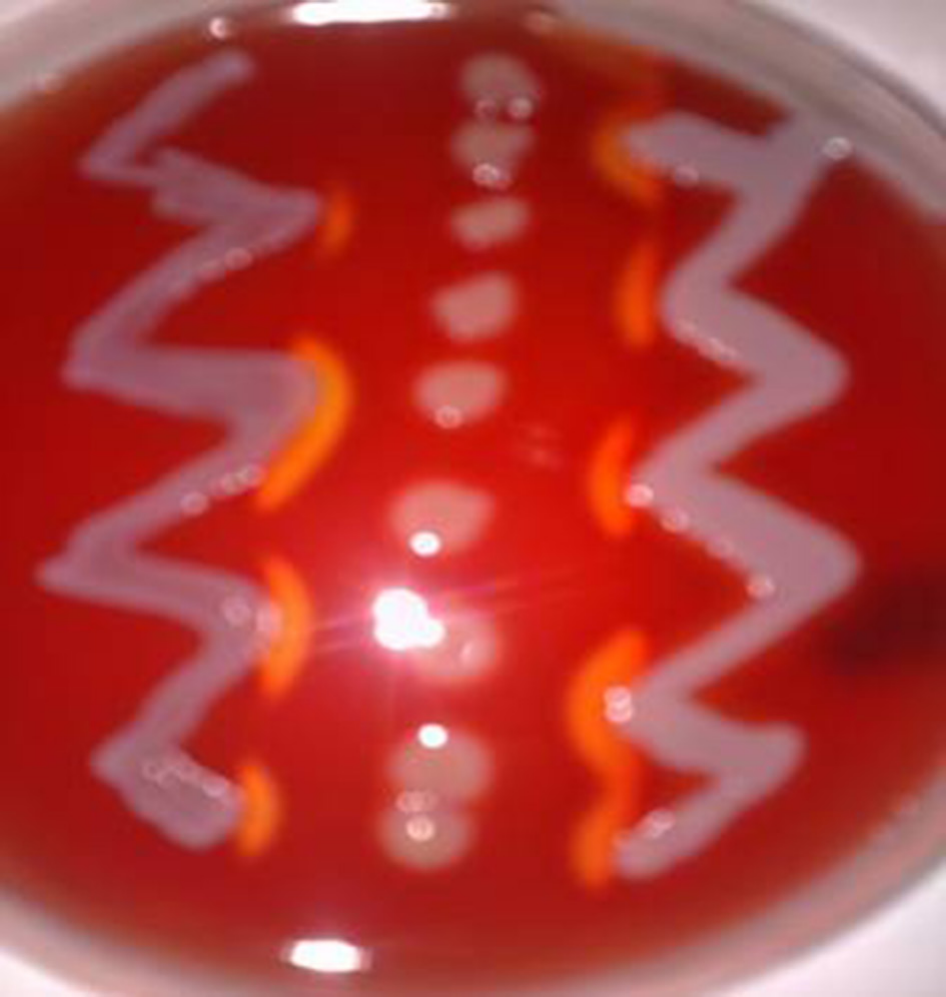
Delta-Hemolysin Detection Beta-hemolytic *S.* *aureus* is seeded vertically and the samples to be tested, were streaked in two sides .Both samples are positive, photo is taken just after incubation and before keeping 4 to 6 hours at room temperature.

The hemolytic activity was evaluated by plating Staphylococci strains on 5% bovine blood for alpha- and beta-hemolysin production. The criteria for hemolysin identification were: complete lytic zone (transparent) with blurred edges for alpha-hemolysin and incomplete (non-transparent) lytic zone, which became complete with sharp edges after overnight incubation at 4 °C, for beta-hemolysin (5). Delta-hemolysin was determined by using the synergistic hemolysis method described by Hebert and Hancock (6) (Figure 1). 

### 3.4. Susceptibility Testing

For susceptibility testing, isolates were incubated in tryptic soy broth at 37 °C for 24 h and the suspension was adjusted to a turbidity equivalent to a 0.5 McFarland standard. Susceptibility to antimicrobial agents was determined for isolated strains by the disk diffusion method on Mueller-Hinton (MH) agar, (Merck, Germany), following the Clinical and Laboratory Standards Institute (CLSI) guidelines (7). The selected antibiotics for antibiogram were Oxacillin, Penicillin, Ciprofloxacin, Erythromycin, Methicillin, Azithromycin, Ofloxacin, Clindamycin and Lincospectin. Isolates were categorized as susceptible and resistant based upon interpretive criteria developed by the (CLSI) (7).

### 3.5. Biofilm and Beta- Lactamase Assays

Beta-lactamase production was detected by test tube iodometric technique as described by Sykes and Mathew (8). The biofilm assay was performed by using microtiter plates as described by Tendolkar et al (9). Interpretation of biofilm production was according to the criteria described by Stepanovic et al (10). Based on these criteria, ODc (optical density cut-off value) is defined as: average OD of negative control + 3 × SD (standard deviation) of negative control, and the biofilms producers are categorized as: no biofilm producer ≤ ODc, weak biofilm producer ODc< ~ ≤ 2 × ODc, moderate biofilm producer 2 × ODc< ~ ≤ 4 × ODc and strong biofilm producer > 4 × ODc. While “~ “ stands for average of sample ODs.

## 4. Results

From sixty one wound infections, 40 isolates (87%) of *S. aureus* and 6 coagulase- negative Staphylococci (CNS) (13%) were recovered, the numbers from dermatitis lesions were 9 (31%) and 20 (69%), respectively. Details are summarized in Table 1

**Table 1.** Isolated Staphylococci From Wound Infections and Various Skin Dermatitis ^a^

Sample	No.	CNS ^b^	*S. aureus*	Total
**Skin dermatitis**	39	20 (69)	9 (31)	29 (100)
**Wound infections**	61	6 (13)	40 (87)	46 (100)
**Total**	100	26 (34.7)	49 (65.3)	75 (100)

^a^ Data are presented as No. (%).

^b^ Abbreviation: CNS, coagulase- negative Staphylococci.

In total, 20 (40.8%), 20 (40.8%), 6 (12.25) and 6 (12.25%) of *S. aureus* isolates were positives for α, β, ∂ and combined αβ-hemolysin productions, respectively. For CNS isolates, only 17 (65.4%) were hemolysin producers that all were ∂ type. Forty one (83.7%) of our *S. aureus* and 19 (73.1%) of CNS isolates were biofilm producers, respectively. The data for β-lactamase production were 14 (28.6) for *S. aureus* and 26 (100%) for CNS isolates. In total, sixty out of 75 (80%) isolated Staphylococci were biofilm producers, out of them 27 isolates (45%) were positive in beta- lactamase test. Table 2 shows the result of overall antimicrobial susceptibility patterns irrespective of ß-lactamase production. The *S. aureus* isolates from wound infections show a high sensitivity pattern to all examined antibiotics, this sensitivity pattern for isolates from skin infections is relatively low, though. 

Excluding *S. aureus* isolates from wound infection, all isolates of Staphylococci show a low sensitivity to methicillin.

**Table 2.** Antibiotic Susceptibility Responses of Isolated Staphylococci From Wound Infections and Various Skin Dermatitis Irrespective of ß-lactamase Production ^a^,^b^

Sample	Wound Infection				Skin Infection			
Staphylococci	*S. aureus*		CNS		*S. aureus*		CNS	
	S	R	S	R	S	R	S	R
**Antibiotics**								
Oxacillin	31 (77.5)	9 (22.5)	4 (66.6)	2 (33.3)	5 (55.6)	4 (44.4)	2 (10)	18 (90)
Penicillin	35 (87.5)	5 (12.5)	1 (16.6)	5 (83.33)	4 (44.4)	5 (12.5)	2 (10)	18 (90)
Methicillin	33 (82.5)	7 (17.5)	2 (33.3)	4 (66.7 )	3 (33.3)	6 (66.7)	1 (5)	19 (95)
Ofloxacin	35 (87.5)	5 (12.5)	5 (83.3)	1 (16.7)	2 (22.2)	7 (77.8)	4 (20)	16 (80)
Ciprofloxacin	37 (92.5)	3 (7.5)	6 (100)	0 (0.0)	6 (66.7)	3 (33.3)	17 (85)	3 (15)
Erythromycin	38 (95)	2 (5)	2 (33.3)	4 (66.7)	5 (556)	4 (44.4)	8 (40)	12 (60)
Azithromycin	34 (85)	6 (15)	2 (33.3)	4 (66.7)	5 (55.6)	4 (44.4)	8 (40)	12 (60)
Clindamycin	36 (90)	4 (10)	4 (66.7)	2 (33.3)	3 (33.3)	6 (66.7)	13 (65)	7 (35)
Lincospectin	37 (93)	3 (8)	4 (66.7)	2 (33.3)	6 (66.7)	3 (33.3)	18 (90)	2 (10)

^a^ Abbreviations: CNS, coagulase- negative Staphylococci; S, sensitive; R, resistant.

^b^ Data are presented as No. (%).

## 5. Discussion

Skin and soft tissue infections (SSTIs) are ubiquitous and the most common of infections. The vast majority of SSTIs are caused by Staphylococci (11). In the present study, the frequency of *S. aureus* causing skin infection is more than CNS, 65.35% and 34.7%, respectively. This is in line with the previous findings of Nishijim et al. (12) and Schmidt et al (13). In total, sixty out of 75 (80%) isolated Staphylococci were biofilm producers. This phenomenon can have deleterious effects because biofilm formation is thought to play an important role in the survival of virulent strains of Staphylococci. In human medicine, it has been estimated that most of nosocomial infections are biofilm associated (14), Moreover, biofilm formation has been shown to be positively correlated with resistance to antimicrobial agents (15). About half (45%) of our biofilm producer isolates were also positive in beta -lactamase test.

Two overall prevalence of 28.5% and 100% of ß-lactamase producers were recorded for *S. aureus* and CNS isolates, respectively, the value for *S. aureus* is lower than about 80% reported by Akindele et al. (16) and Efuntoye and Amuzat (17) but higher than about 86% for CNS reported by Habeeb and Mohammad (18). ß-lactamase production by staphylococci is the recognized mechanism of resistance to ß-lactam antibiotics, such as penicillin G, methicillin and ampicillin, as such the low prevalence of ß-lactamase production by *S. aureus* isolated from wound infections (20%), and high prevalence (66.6%), from skin dermatitis, explains the high and low sensitivity pattern of *S. aureus* isolates from wound infection and dermatitis lesions to examined ß-lactam antibiotics, respectively. This suggests that the ß-lactamase resistant anti-staphylococcal agents should be selected as a first choice to treat dermatitis lesions.

All CNS isolates were positive for ß-lactamase production test, a low sensitivity pattern of CNS for oxacillin, penicillin and methicillin was shown in Table 2 irrespective of their origin. Excluding *S. aureus* isolates from wound infections, all isolates of Staphylococci show a high resistance rate (66%-95%) to methicillin. These results are compatible with some reports that indicate a rate of 40% -96% of MRSA from several recent studies in Iran (19). It is documented that with the frequent antimicrobial treatment prescribed for dermatitis patients, methicillin-sensitive *S. aureus* colonies present on skin lesions, are often replaced by MRSA (20). The ratio of isolation of (MRSA) strains appears to have decreasing in skin infections in some countries (19) and increasing in some others (21). The relative high sensitivities of our *S. aureus* isolates from wound infections to most examined antimicrobials are in agree with Japoni et al. report from Shiraz, Iran (22).

Twenty out of 49 (40.8%), of our *S. aureus* isolates were α-haemolysin producers, out of them, 19 (95%) were biofilm producers simultaneously. Caiazza and Toole (23) showed a role for α-hemolysin in *S. aureus* biofilm formation and that this toxin appears to be required for cell-to-cell interactions. The frequency of β-haemolysin producer isolates is such as α- hemolysin producers, the role of beta-hemolysin in disease is not clearly understood. It is not dermonecrotic in guinea pigs, and it is not lethal in mice. It was found to have a phosphorylase C activity (24).

Six out of 49 (12.25%) of our *S. aureus* isolates were ∂-haemolysin producers, This is much lower from reports that recorded 80%- 97%∂-hemolysin production by *S. aureus* isolates (24). This toxinis capable of causing membrane damage in a variety of mammalian cells, as well as subcellular structures such as membrane-bound organelles, spheroplasts and protoplasts (25).

Sakoulas et al. reported absence of delta-hemolysin expression in *S. aureus* isolates suggestive of suppression of relative gene function in these isolates (26). Twenty two out of 26 (84.6%) isolates of CNS were hemolysin producers that all were ∂ type. Reports estimated a 40% - 80% of CNS have the ability to produce this toxin (24, 27). CNS strains able to produce ∂-hemolysin, were isolated from infectious processes of newborns in hospitals (28) . 

In conclusion, the high percentage of hemolysins, biofilm and beta- lactamase production by isolated Staphylococci obtained in this work, suggests, an important role of these virulence factors in the pathogenesis of isolated Staphylococci from dermatitis lesions. The presence of two or more virulence factors could increase the pathogenic ability of isolates in relation to those that express only one virulence factor, however, further research should be performed. The *S. aureus* isolates from wound infections show a high sensitivity pattern to all examined antibiotics. Ciprofloxacin was found to be active Butmethicillin followed by Ofloxacin, were found to be low active drugs against isolates from dermatitis lesions.

## References

[1] Stevens DL, Bisno AL, Chambers HF, Everett ED, Dellinger P, Goldstein EJ (2005). Practice guidelines for the diagnosis and management of skin and soft-tissue infections.. Clin Infect Dis..

[2] Del Giudice P, Blanc V, Durupt F, Bes M, Martinez JP, Counillon E (2006). Emergence of two populations of methicillin-resistant Staphylococcus aureus with distinct epidemiological, clinical and biological features, isolated from patients with community-acquired skin infections.. Br J Dermatol..

[3] Vandecasteele SJ, Peetermans WE, R. Merckx R, Rijnders BJ, Van Eldere J (2003). Reliability of the ica, aap and atlE genes in the discrimination between invasive, colonizing and contaminant Staphylococcus epidermidis isolates in the diagnosis of catheter-related infections.. Clin Microbiol Infect..

[4] Hall-Stoodley L, Stoodley P (2002). Developmental regulation of microbial biofilms.. Curr Opin Biotechnol..

[5] Murray PR, Baron EJ, Jorgensen JH, Landry ML, Pfaller MA, Yolken RH (2003). Manual of Clinical Microbiology..

[6] Hebert GA, Hancock GA (1985). Synergistic hemolysis exhibited by species of staphylococci.. J Clin Microbiol..

[7] Franklin R, Cockerill III (2011). Performance Standards for Antimicrobial Susceptibility Testing; Twenty First Informational Supplement, M100S21..

[8] Skyes RB, Mathews M, Hamilton-Miller JMT, Smith JT (1979). Detection assay and immunology of beta-lactamases.. Beta Lactamases..

[9] Tendolkar PM, Baghdayan AS, Gilmore MS, Shankar N (2004). Enterococcal surface protein, Esp, enhances biofilm formation by Enterococcus faecalis.. Infect Immun..

[10] Stepanovic S, Vukovic D, Hola V, Di Bonaventura G, Djukic S, Cirkovic I (2007). Quantification of biofilm in microtiter plates: overview of testing conditions and practical recommendations for assessment of biofilm production by staphylococci.. APMIS..

[11] Dryden MS (2010). Complicated skin and soft tissue infection.. J Antimicrob Chemother..

[12] Nishijim S, Ohshima S, Higashida T, Nakaya H, Kurokawa I (2003). Antimicrobial resistance of Staphylococcus aureus isolated from impetigo patients between 1994 and 2000.. Int J Dermatol..

[13] Schmidt-Ioanas M, de Roux A, Lode H (2005). New antibiotics for the treatment of severe staphylococcal infection in the critically ill patient.. Curr Opin Crit Care..

[14] Singhai M, Malik A, Shahid M, Malik A, Rawat V (2012). Colonization of peripheral intravascular catheters with biofilm producing microbes: Evaluation of risk factors.. Niger Med J..

[15] Moretro T, Hermansen L, Holck AL, Sidhu MS, Rudi K, Langsrud S (2003). Biofilm formation and the presence of the intercellular adhesion locus ica among staphylococci from food and food processing environments.. Appl Environ Microbiol..

[16] Akindele AA, Adewuyi IK, Adefioye OA, Adedokun SA, Olaolu AO (2010). Antibiogram and beta-lactamase production of Staphylococcus aureus isolates from different human clinical specimens in a tertiary health institution in Ile-ife, Nigeria.. Am-Eurasian J Sci Res..

[17] Efuntoye MO, Amuzat MA (2007). Beta Lactamase production by Staphylococcus aureus from children with sporadic Diarrhoea in Ibadan and Ago-Iwoye, Nigeria.. Afr J Biomed Res..

[18] Khadri H, Alzohairy M (2010). Prevalence and antibiotic susceptibility pattern of methicillin-resistant and coagulase-negative staphylococci in a tertiary care hospital in India.. Int J Med Med Sci..

[19] Khalili H, Dashti-Khavidaki S, Karimzadeh I, Jafari S, Abdollahi A, Shahidi MR (2012). Changes in 4-year antimicrobial resistance pattern of gram-positive bacteria at the main referral teaching hospital, Tehran, Iran.. Acta Med Iran..

[20] Rahimi F, Bouzari M, Katouli M, Pourshafie M (2013). Prophage Typing of Methicillin Resistant Staphylococcus aureus Isolated from a Tertiary Care Hospital in Tehran, Iran.. Jundishapur J Microbiol..

[21] Tada J, Yamasaki H, Toi Y, Akiyama H, Arata J (1999). Is the face and neck pattern of atopic dermatitis in Japan a special variant?. Am J Contact Dermat..

[22] Japoni A, Ziyaeyan M, Jmalidoust M, Farshad S, Alborzi A, Rafaatpour N (2010). Antibacterial susceptibility patterns and cross-resistance of methicillin resistant and sensitive staphyloccus aureus isolated from the hospitalized patients in shiraz, iran.. Braz J Microbiol..

[23] Caiazza NC, O'Toole GA (2003). Alpha-toxin is required for biofilm formation by Staphylococcus aureus.. J Bacteriol..

[24] Dinges MM, Orwin PM, Schlievert PM (2000). Exotoxins of Staphylococcus aureus.. Clin Microbiol Rev..

[25] Verdon J, Girardin N, Lacombe C, Berjeaud JM, Hechard Y (2009). delta-hemolysin, an update on a membrane-interacting peptide.. Peptides..

[26] Sakoulas G, Eliopoulos GM, Moellering RC, Jr., Wennersten C, Venkataraman L, Novick RP (2002). Accessory gene regulator (agr) locus in geographically diverse Staphylococcus aureus isolates with reduced susceptibility to vancomycin.. Antimicrob Agents Chemother..

[27] Hebert GA (1990). Hemolysins and other characteristics that help differentiate and biotype Staphylococcus lugdunensis and Staphylococcus schleiferi.. J Clin Microbiol..

[28] Cunha Mde L, Rugolo LM, Lopes CA (2006). Study of virulence factors in coagulase-negative staphylococci isolated from newborns.. Mem Inst Oswaldo Cruz..

